# Development and identification of an elite wheat-*Hordeum californicum* T6HcS/6BL translocation line ND646 containing several desirable traits

**DOI:** 10.1590/1678-4685-GMB-2022-0117

**Published:** 2022-10-07

**Authors:** Zhangjun Wang, Qingfeng Li, Caixia Liu, Fenglou Liu, Nali Xu, Mingming Yao, Huixia Yu, Yanqing Wang, Jiajing Chen, Shuangyu Bai, Jingxin Yang, Gang Sun, Jiaohui Long, Yalei Fan, Ling Kang, Hongxia Li, Xiaogang Zhang, Shengxiang Liu

**Affiliations:** 1 Nanjing Agricultural University, Cytogenetics Institute, State Key Laboratory of Crop Genetics and Germplasm Enhancement, Nanjing, Jiangsu, China.; 2 Ningxia University, School of Agriculture, Yinchuan, Ningxia, China.; 3 Ningxia Academy of Agricultural-Forestry Sciences, Institute of Crop Sciences, Yinchuan, Ningxia, China.

**Keywords:** Wheat, H. californicum, translocation line, molecular identification, multiple desirable agronomic trait

## Abstract

*Hordeum californicum* (*H. californicum*, 2n=2X=14, H^c^H^c^), one of the wild relatives of wheat (*Triticum aestivum* L.), harbors many desirable genes and is a potential genetic resource for wheat improvement. In this study, an elite line ND646 was selected from a BC_4_F_5_ population, which was developed using ^60^Co-γ irradiated wheat-*H. californicum* disomic addition line WJ28-1 (DA6H^c^) as the donor parent and Ningchun 4 as the recurrent parent. ND646 was identified as a novel wheat-*H. californicum* 6H^c^S/6BL translocation line using genomic *in situ* hybridization (GISH), fluorescence *in situ* hybridization (FISH), and *H. californicum*-specific expressed sequence tag (EST) markers. Further evaluation revealed that ND646 had excellent performance in several traits, such as a higher sedimentation value (SV), higher water absorption rate (WAR), and higher hardness index (HI). More importantly, it had more kernels per spike (KPS), a higher grain yields (GY), and good resistance to powdery mildew, leaf rust, and 2,4-D butylate (2,4-D). Its excellent phenotypic performance laid the foundation for further investigation of its genetic architecture and makes ND646 a useful germplasm resource for wheat breeding.

## Introduction

Wheat (*Triticum aestivum* L.) is one of the most important crops in the world and supplies food to at least one-third of the global population (Yang et al., 2016). However, the narrow genetic basis has resulted in a bottleneck in wheat breeding (Gupta et al., 2010). Therefore, increasing genetic variation is urgently needed for wheat improvement. Wild relatives of wheat, carrying significant genetic diversity and numerous desirable characteristics, have played an important role in wheat improvement (Mujeeb-Kazi et al., 2013). One of the most successful Chinese wheat cultivars, ‘Xiaoyan 6’ was created by the hybridization between common wheat and *Elytrigia elongate* (Li et al., 1990). To date, many desirable genes have been successfully introduced from wild relatives into common wheat. The wheat-rye T1RS/1BL translocations carrying disease resistance genes *Pm8*, *Yr9*, *Lr26*, and *Sr31*, as well as genes associated with superior agronomic and abiotic stress traits, have been widely used in wheat breeding (Ren et al., 2017; Howell et al., 2019; McIntosh et al., 2019; Li *et al.*, 2020; Zhou et al., 2021). Powdery mildew resistance genes *Pm21* and *Pm67* from *Haynaldia villosa* (Xing et al., 2018; Zhang et al., 2021), and the Fusarium head blight resistance gene *Fhb7* from *Thinopyrum elongatum* also contributed significantly to wheat genetic improvement (Wang et al., 2020).

*Hordeum* ssp. *californicum* (*H. californicum*), one of the most important wild relatives of wheat, has been valued as a donor of important agronomic and resistance traits, including tolerance to cold and low-nitrogen conditions, resistance to powdery mildew and leaf rust, more kernels per spike (KPS), and higher grain weight (Gupta and Fedak, 1985; Armstrong et al., 1993; Kolb et al., 2002; Kong et al., 2007; Fang et al., 2014; Mehnaz et al., 2021). To introduce favorable genes underlying the above traits into wheat, the F_1_ hybrid and amphiploid of intergeneric hybridization between *H. californicum* and commonly wheat cv. ‘Chinese Spring’ (CS) were acquired through a wide cross, (Gupta and Fedak, 1985). A series of wheat-*H. californicum* chromosome lines, including five disomic addition lines (DA2H^c^, DA3H^c^, DA4H^c^, DA6H^c^, and DA7H^c^) and one disomic substitution line (DS6H^c^), were obtained (Gupta and Fedak, 1985; Fang *et al.*, 2014). In addition, 303 markers were assigned to chromosomes 1H^c^-7H^c^ to identify the homoeologous groups of the alien chromosome in wheat-*H. californicum* derived materials and to trace the alien chromosomes (Kong *et al.*, 2008; Fang *et al.*, 2014). This study identified a wheat-*H. californicum* translocation line, ND646, by molecular cytogenetics and evaluated its phenotypic performances in multiple important traits. This research provides a novel germplasm resource for wheat breeding.

## Material and Methods

### Material

Wheat cv. CS-*H. californicum* amphiploid (2n=56, genome AABBDDH^c^H^c^) (Accession No. TA3443) was kindly provided by the Wheat Genetics and Genomics Resource Center, Kansas State University, Manhattan, Kansas, USA. Common wheat cv. CS, ‘Ningchun 4’, and ‘Hongtuzi’ were maintained by the Key Laboratory of Modern Molecular Breeding for Dominant and Special Crops in Yinchuan, Ningxia, China. WJ28-1 is a disomic addition line developed from a BC_1_F_3_ population made with CS as the recurrent parent and *H. californicum* amphiploid as the donor parent. WJ28-1 contains several excellent traits, such as more kernels per spike (KPS), higher grain yield (GY), and good resistance to powdery mildew.

### Development of the wheat-*H.californicum* translocation line

The pollen of WJ28-1 plants at the booting stage were irradiated with ^60^Co-γ ray at a dosage of 20 Gray (Gy) and a dose rate of 0.5 Gy·min^-1^ (Song et al., 2013). The irradiated pollen was pollinated to an emasculated Ningchun 4. Then M_1_ seeds were backcrossed with Ningchun 4 or self-pollinated for several generations until stable agronomic performances were obtained. Meanwhile, their progeny were characterized by genomic *in situ* hybridization (GISH), fluorescence *in situ* hybridization (FISH), and their molecular markers.

### GISH and FISH analysis

Metaphase I (MI) chromosome spreads from root-tip cells and pollen mother cells (PMCs) were prepared as described by Gill et al. (1991). Total genomic DNA was extracted following the method of Yang et al. (2006). GISH was performed according to the protocols described by Jiang and Gill (1993). FISH was performed according to the method of Jiang *et al.* (1994). The oligonucleotide probes pSc119.2 and pAs1 were labeled with biotin-16-dUTP and digoxigenin-11-dUTP, respectively (McIntyre et al., 1990; Nagaki et al., 1995; Du et al., 2017). Anti-digoxigenin-rhodamine Fab fragments (Roche Diagnostics GmbH, Germany) and streptavidin-fluorescein thiocyanate (FITC) (Roche Diagnostics GmbH, Germany) were used, followed by staining with 4,6-al amidine-2-phenyl indole (DAPI) to detect digoxigenin and biotin signals, respectively. Signals were visualized under an Olympus BX60 Fluorescence microscope (Olympus Optical Co. Ltd, Tokyo, Japan). Images were captured with a SPOT 32 CCD camera (SPOT Charge Coupled Device, Diagnostic Instruments, Inc., Sterling Heights, MI, USA) and analyzed using Adobe Photoshop software.

### Molecular marker analysis

Four expressed sequence tag PCR (EST-PCR) primer pairs were used to identify the origin of the *H. californicum* chromosome in ND646 (Fang et al., 2014). Information on 6H^c^-specific markers is shown in Table 1. The EST-PCR program was one cycle at 94 °C for 3 min, followed by 32 cycles of 94 °C for 30 s, at annealing temperature at 55 °C for 45 s, and at 72 °C for 1 min, then a final extension at 72 °C for 10 min. The amplified PCR products were separated by polyacrylamide gel electrophoresis (PAGE) with an acrylamide concentration of 8% and stained with silver (Bassam and Gresshoff, 2007).

**Table 1 -:** Detailed information of specific molecular markers of *H.californicum*.

Marker	EST ID	Chromosome location	Primer squence (5’-3’)	Arm of chromosome
*CINAU91*	BF145253	6AS 0.65-1.00 6DS 0.79-0.99	L: CCTCGTGGAGGAGAACTTCA R: GTGACCATGTCGGTGAACTG	6H^c^S
*CINAU502*	Ta#S52546774	6AS 0.35-0.65 6DS 0.99-1.00	L: TTTTTCAGTGGAGGGGTCAC R: GACGGCGACTGGTTGTTAAT	6H^c^S
*CINAU506*	BE591788	6AL 0.90-1.00 6BL 0.36-0.40 6DL 0.74-0.80	L: ATGGAGAGAGCGCTGTAATA R: CCCCTACATGAAATGAGAAG	6H^c^L
*CINAU511*	BE425153	6AL 0.90-1.00 6BL 0.40-1.00 6DL 0.47-0.68	L: GAACATAGCCGAAGCATTAC R: CTCTACCTGGGCTACTCCTT	6H^c^L

### Experimental design

Field experiments were conducted at the teaching experiment farm of Ningxia University (Regional trial Ⅰ) and the research station of the Institute of Crop Science, Ningxia Academy of Agricultural-Forestry Sciences at Yongning, Ningxia (Regional trial Ⅱ) in 2021. ND646 and Ningchun 4 were randomized at each location as a complete block design with three replications. The plot size was 12.87 m^2^. The plots were spaced 0.15 m apart. For each plot, approximately 3000 seeds were evenly planted. Grain yield-related traits were evaluated at both locations. All the other traits, including disease and herbicide resistance, and grain quality traits, were only evaluated at the teaching experiment farm of Ningxia University.

### Disease and herbicide resistance evaluation

To evaluate powdery mildew resistance, a mixture of prevalent spore isolates of *Blumeria graminis* f. sp. *tritici* from Ningxia, China, was used to infect the susceptible wheat cultivar Hongtuzi and the experimental plants at the elongation stage. The disease grades were scored as 0 (immune, I), 0-1 (nearly immune, NI), 1-2 (highly resistant, HR), 3-4 (moderately resistant, MR), 5-6 (moderately susceptible, MS), 7-8 (highly susceptible, HS), and 9 (extremely susceptible, ES), according to Sheng and Duan (1991). For leaf rust resistance evaluation, mixed *Puccinia recondita* f. sp. *tritici* isolates was used to infect plants at the elongation stage, and the disease responses were recorded with a 0-4 rating, in which 0-2 was considered resistant and 3-4 was considered susceptible (Roelfs et al., 1992). For herbicide resistance evaluation, 0.8% 2, 4-D was sprayed onto the leaves of wheat at the elongation and heading stages.

### Agronomic and grain quality traits, and grain filling characteristics

During the growing season, twenty spikes from 10 individual plants were randomly selected in each plot before 9:00 a.m. every five days from flowering to maturity. Grains were used to measure fresh grain weight (FGW), grain volume (GV), dry grain weight (DGW), and grain moisture (GM). The grain filling rate (GFR) was calculated according to Wang et al. (2016). After harvest, all materials were measured for agronomic traits, including fertile tiller number per plant (FTNPP), plant height (PH), spike length (SL), spikelets per spike (SPS), fertile spikelets per spike (FSPS), grain weight per spike (GWPS), biological yield (BY), economic yield (EY), economic coefficient (EC), kernels per spike (KPS) with ten replications and fertile spike number (FSN), thousand-kernel weight (TKW), and grain yield (GY) with three replications. A sample of 30 g of grain was used to measure grain quality traits, including moisture content (MC), crude protein content (CPC), wet gluten content (WGC), flour yield rate (FYR), sedimentation value (SV), falling number (FN), water absorption rate (WAR), hardness index (HI), stabilization time (ST), formation time (FT), volume weight (VW) and extension area (EA), with three replications by Near Infra Red Spectrum (NIRS) DA7200 (Perten, Sweden). Statistical analyses were conducted in Excel 2010 and DPS 7.05.

## Results

### Development of ND646

**Figure 1 -:**
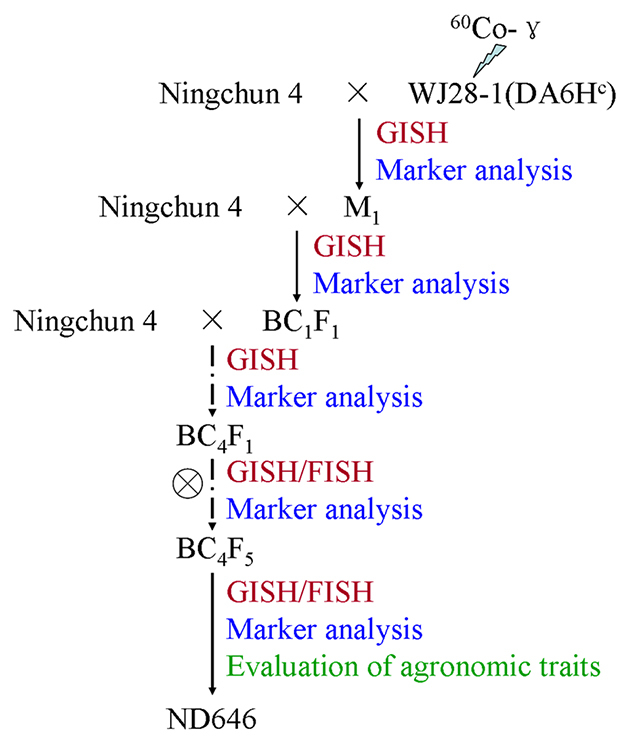
Scheme of the development of whea**-**t*H.californicum* translocation line ND646.

In previous work, the wheat-*H. californicum* disomic addition line WJ28-1 showed excellent performance for several traits, including disease resistance, more spikelets per spike, and more kernels per spike. To create wheat-*H. californicum* translocation lines with small alien chromosome fragments, we irradiated the pollen of WJ28-1 with ^60^Co-γ and pollinated them to an emasculated cultivar, ‘Ningchun 4’. The progeny were backcrossed to ‘Ningchun 4’ for three generations to produce BC_4_F_1_ seeds. BC_4_F_1_ was then crossed for four generations to produce BC_4_F_5_ seeds. For each generation of back-crossing and self-crossing, the progeny were characterized by GISH/FISH and 6H^c^ specific EST markers. ND646 is an elite line selected from the BC_4_F_5_ population (Figure 1).

### Characterization of ND646 by sequential GISH/FISH and molecular markers

**Figure 2 -:**
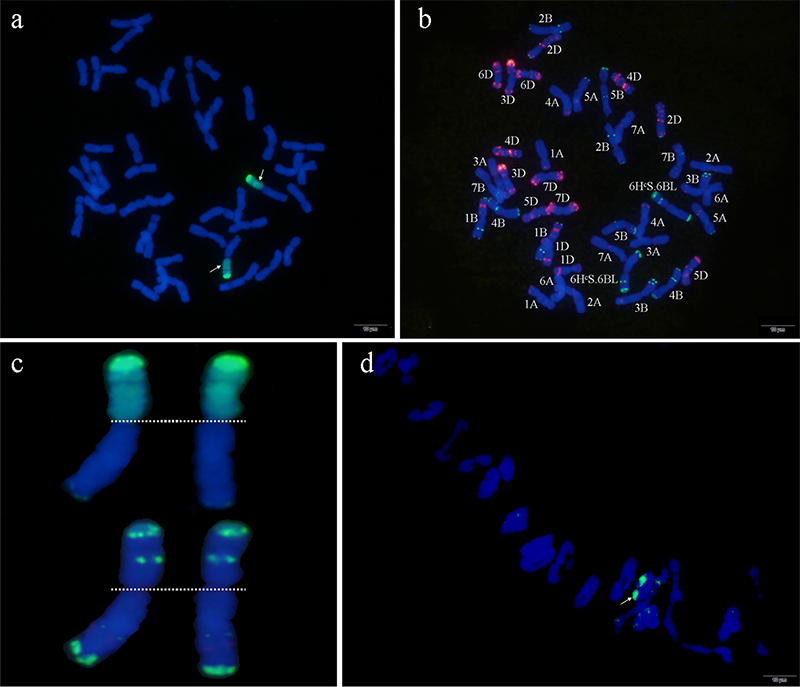
Sequential GISH/FISH analysis of the wheat**-** *H.californicum* translocation line ND646. a. GISH of ND646, the genome DNA of *H.californicum* was visualized in bright green; b. FISH of ND646 using pSc119.2 (shown in red) and pAs1 (shown in green) repetitive DNA as probes, white arrows show the translocation chromosome; c. GISH and FISH diagram of translocation chromosomes in DN646, respectively; d. There were 21 bivalents containing one translocation bivalent (shown in green) at the metaphase I (MI) stage in pollen mother cells (PMCs) of ND646 (Scale bar = 20µm).

ND646, selected from the BC_4_F_5_ (Ningchun 4 × ^60^Co-γ irradiated WJ28-1), was analyzed using GISH/FISH. Based on the molecular karyotype of *H. californicum* and common wheat (Mukai et al., 1993; Fang et al., 2014; Du et al., 2017), ND646 was identified as a 6H^c^S/6BL translocation line (2n = 42) (Figure 2a-c). Meiosis in the PMCs of ND646 and its self-crossed progeny were investigated. Among the 100 PMCs at the metaphase I stage showing 2n = 21, all cells had one translocation bivalent (Figure 2d), and each cell contained 0.06 univalents, 3.92 rod bivalents, and 17.05 ring bivalents, on average. Thus, ND646 is a cytogenetically stable wheat-*H. californicum* translocation line.

**Figure 3 -:**
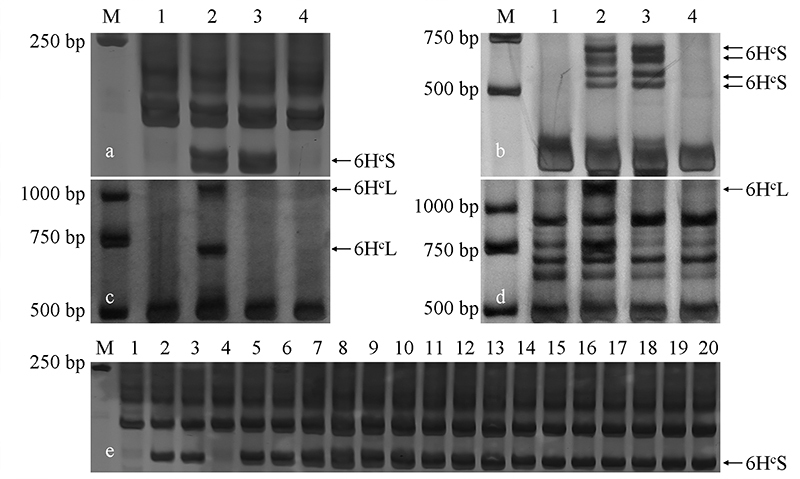
Amplification of EST-PCR primer pairs in the parents and ND646. a**-**d. Markers CINAU 91, CINAU 502, CINAU 506 and CINAU 511, respectively. 1**-**4 were CS, WJ28-1, ND646 and Ningchun 4, respectively; 5**-**20 were the amplification of marker CINAU 91 in 16 individual plants of ND646; M was a DNA marker with a molecular weight of 100, 250, 500, 750, 1000 and 2000 bp. Arrows showed specific banding of 6H^c^S and 6H^c^L chromosomes.

The GISH and FISH results indicated that the alien chromosome fragment of ND646 is 6H^c^. To further verify the origin of alien chromosome fragments, 6H^c^-specific markers were used to amplify the total genomic DNA of CS, WJ28-1, ND646, and Ningchun 4. All the ND646 plants could be amplified by the 6H^c^ short-arm specific markers but not by the 6H^c^ long-arm specific markers (Figure 3). Thus, the alien chromosome fragments of ND646 were confirmed as the short arm of chromosome 6H^c^.

### Agronomic, grain yield, and grain quality traits of ND646

The agronomic traits, including SL (11.83 cm vs. 10.92 cm), SPS (21.50 vs. 20.20), and GWPS (2.78 g vs. 2.04 g) in regional trial I. KPS (61.10 vs. 44.50 in regional trial I and 49.51 vs. 43.78 in regional trial 2), and GY (8614 kg.ha^-1^ vs. 7974 kg.ha^-1^ in regional trial I and 11093 kg.ha^-1^ vs. 10417 kg.ha^-1^ in regional trial II) were significantly (p < 0.05) higher in ND646 than in Ningchun 4 (Table 2). The grain quality traits, including SV (30.17 mL vs. 26.94 mL), WAR (59.94% vs. 58.81%) and HI (69.27 vs. 63.69), were also significantly (p < 0.05) higher in ND646 than in Ningchun 4 in regional trial I (Table 2).

**Table 2 -:** Performances on agronomic, grain yield and grain quality traits of Ningchun 4 and ND646.

Traits	Index	Ningchun 4	ND646
Agronomic	FTNPP (No.)	2.30±0.675	2.60±0.843
	PH (cm)	91.07±2.948	93.25±0.674
	SL (cm)	10.92±0.585	11.83±0.897 ^*^
	SPS (No.)	20.20±1.549	21.50±0.707 ^*^
	FSPS (No.)	18.50±1.581	19.30±1.636
	GWPS (g)	2.04±0.433	2.78±0.539 ^*^
	EC	0.45±0.051	0.46±0.041
Yield-related	FSN (million myriad·ha^-1^)	450.56±91.320	506.48±60.266
	KPS (No.)	44.50±10.233	61.10±12.096 ^*^
	TKW (g)	41.56±0.439	42.70±0.453
	TY (kg·ha^-1^)	9307.22±84.374	12372.19±833.186
	GY(kg·ha^-1^)	7974.70±625.512	8614.75±641.201 ^*^
	FSN (million myriad·ha^-1^)	448.89±134.220	462.22±94.595
	KPS (No.)	43.78±6.641	49.51±5.706 ^*^
	TKW (g)	43.48±2.898	46.54±3.850
	TY (kg·ha^-1^)	8597.30±3257.577	10533.02±1625.845
	GY(kg·ha^-1^)	10417.97±93.869	11093.65±18.894 ^*^
Grain Quality	CPC (%)	14.29±0.053	13.97±0.225
	WGC (%)	29.96±0.265	30.14±0.572
	FYR (%)	67.94±0.414 ^*^	65.09±0.203
	SV (mL)	26.94±0.532	30.17±1.259 ^*^
	FN (s)	448.10±3.779	429.81±6.542
	WAR (%)	58.81±0.555	59.94±0.303 ^*^
	HI	63.69±1.529	69.27±0.451 ^*^
	ST (min)	3.53±0.365	5.03±0.820
	FT (min)	3.76±0.302	4.15±0.078
	VW (g·L^-1^)	818.32±0.894 ^*^	793.09±1.203
	EA (cm^2^)	103.04±6.904	109.18±16.620

PTNPP=Fertile tiller number per plant, PH=Plant height, SL=Spike length, SPS=Spikelets per spike, FSPS=Fertile spikelets per spike, GWPS=Grain weight per spike, EC=Economic coefficient, FSN=Fertile spike number, KPS=Kernels per spike, TKW=Thousand kernel weight, TY=Theoretical yield, GY=Grain yield, CPC=Crude protein content, WGC=Wet gluten content, FYR=Flour yield rate, SV=Sedimentation value, FN=Falling number, WAR=Water absorption rate, HI=Hardness index, ST=Stabilization time, FT=Formation time, VW=Volume weight, EA=Extension area. * represented significant differences within the same trait between the two materials (p<0.05).

### Resistance of ND646 to disease and herbicide

**Figure 4 -:**
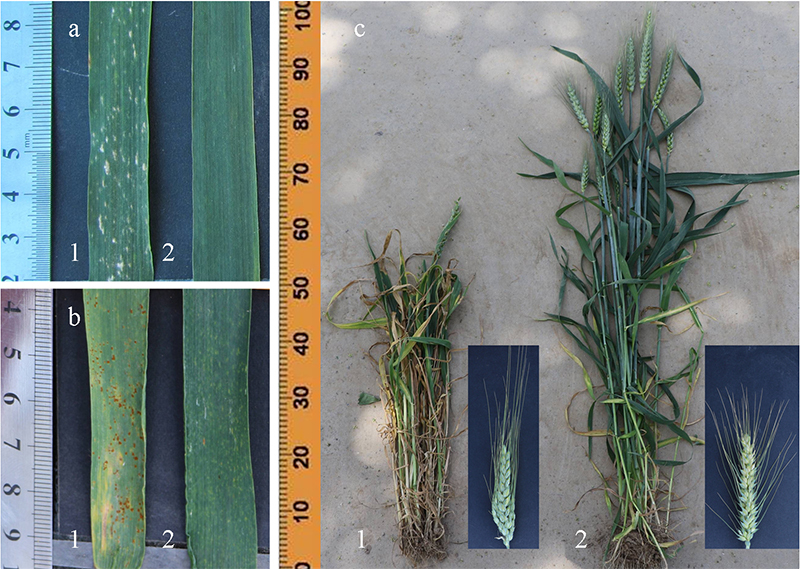
Responses to diseases and herbicides of Ningchun 4 and ND646. 1 and 2 represent Ningchun 4 and ND646, respectively. a. Powdery mildew on flag leaves; b. Leaf rust on flag leaves; c. Effects of 2,4-D on spikes and whole plants, respectively.

Resistance of the adult ND646 plants to powdery mildew, leaf rust, and herbicide were evaluated in the field. ND646 was resistant to powdery mildew, whereas its common wheat parent, Ningchun 4, was susceptible when infected with a mixture of *Blumeria graminis* f. sp. *tritici* isolates (Figure 4a). Furthermore, ND646 had a higher level of resistance to leaf rust than Ningchun 4, with disease ratings of 3 and 2, respectively (Figure 4b). When treated with 2,4-D, the whole plant and spikes of Ningchun 4 were seriously distorted. However, ND646 showed high resistance to 2,4-D and showed robustly growth (Figure 4c). Therefore, ND646 is a novel gene source for improving the disease and herbicide resistance in wheat in Ningxia.

### Grain filling characteristic of ND646

**Figure 5 -:**
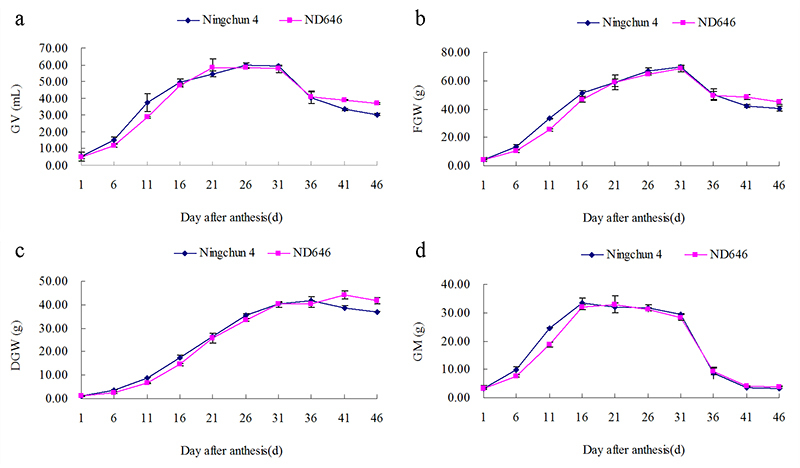
Dynamics of grain volume (GV), fresh grain weight (FGW), dry grain weight (DGW), and grain moisture (GM) of Ningchun 4 and ND646 during grain filling.

In Ningxia, the grain filling of spring wheat occurs from late May to early July. During the late period of grain filling, the temperature could be higher than 25 °C. The grain filling of many wheat cultivars in Ningxia is severely impacted by this high temperature. To investigate the grain filling characteristics of ND646, the dynamics of different morphology traits, including the GV, FGW, DGW, GM, and GFR of ND646 and Ningchun 4, were monitored during grain filling stage. The dynamics of GV, FGW, DGW, and GM of Ningchun 4 and ND646 both showed “S” curves with an increasing trend in the first phase after anthesis followed by a plateau and then a decreasing trend (Figure 5).

**Figure 6 -:**
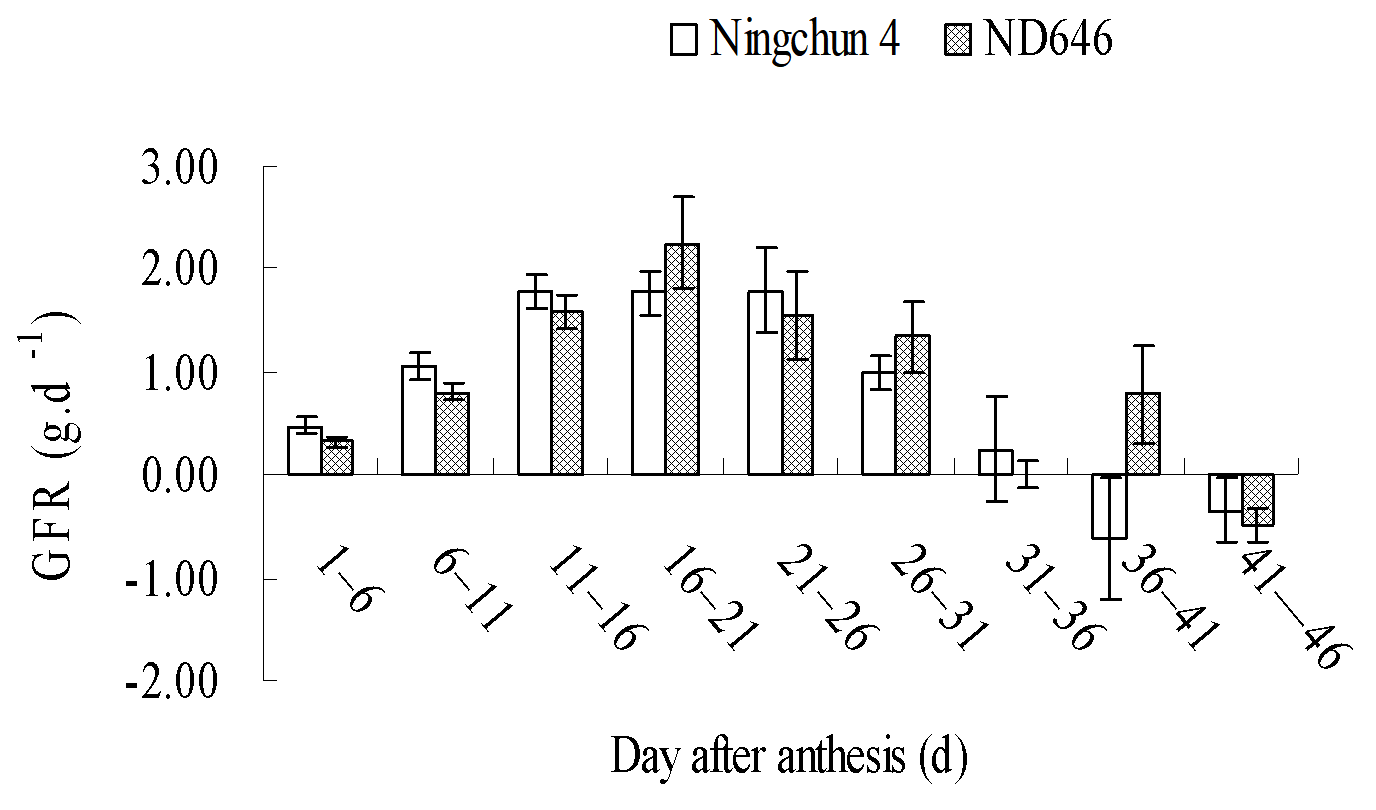
Dynamics of grain filling rate of Ningchun 4 and ND646.

The increases in GV, FGW, and GM during the first 21 days and DGW during the first 36 days after anthesis were higher in ND646 than in Ningchun 4 (Table 3). The GV of Ningchun 4 and ND646 reached a maximum of 59.581 mL on the 26^th^ day and 58.433 mL on the 21^st^ day after anthesis, respectively (Table 3; Figure 5a). The FGW of Ningchun 4 and ND646 reached a maximum of 69.544 g and 68.544 g, respectively, both on the 31^st^ day after anthesis (Table 3; Figure 5b). The DGW of Ningchun 4 and ND646 reached a maximum of 41.513 g on the 36^th^ day and 44.207 g on the 41^st^ day after anthesis, respectively (Table 3; Figure 5c). The GM of Ningchun 4 and ND646 reached a maximum of 33.458 g on the 16^th^ day and 32.824 g on the 21^st^ day after anthesis, respectively (Table 3; Figure 5d). The GFR of Ningchun 4 and ND646 reached the peaks of 1.792 g.d^-1^ on the 21^st^-26^th^ day and 2.255 g.d^-1^ on the 16^th^-21^st^ day, respectively (Table 4; Figure 6). Grain filling of Ningchun4 was completed on the 36^th^-41^st^ day after anthesis, while the grain filling of ND646 was fully completed on the 41^st^-46^th^ day after anthesis. By monitoring the dynamics of grain morphology traits during grain filling, we found that ND646 had a longer grain filling period and a higher maximum GFR at the critical filling stage than Ningchun 4.

**Table 3 -:** Grain morphological characteristics of Ningchun 4 and ND646 at the grain filling stage.

Days after anthesis (d)	GV (mL)		FGW (g)		DGW (g)		GM (g)	
	Ningchun 4	ND646	Ningchun 4	ND646	Ningchun 4	ND646	Ningchun 4	ND646
1	5.281	4.824	4.194	4.137	0.919	0.871	3.275	3.266
6	15.214 ^*^	11.736	13.091 ^*^	10.096	3.320 ^*^	2.476	9.771 ^*^	7.620
11	37.261	28.507	33.103 ^**^	25.127	8.671 ^**^	6.533	24.433 ^**^	18.594
16	49.685	47.462	50.981	46.409	17.523 *	14.444	33.458	31.966
21	54.618	58.433	58.441	58.544	26.365	25.721	32.076	32.824
26	59.581	58.103	66.944	64.557	35.323	33.490	31.621	31.067
31	59.483	57.784	69.544	68.544	40.275	40.213	29.269	28.331
36	40.359	40.597	50.251	49.520	41.513	40.287	8.738	9.233
41	33.304	39.085 ^**^	41.966	48.252 ^**^	38.443	44.207 ^*^	3.523	4.045 ^*^
46	30.353	36.991 **	40.322	44.845 *	36.702	41.702 *	3.143	3.814 *

GV = Grain volume, FGW = Fresh grain weight; DGW = Dry grain weight, GM = Grain moisture. * and ** represent significant (p < 0.05) and highly significant (p < 0.01) differences, respectively, in the corresponding traits between Ningchun 4 and ND646.

**Table 4 -:** Dynamic changes in the grain filling rate of Ningchun 4 and ND646.

Days after anthesis (d)	Ningchun 4	ND646
1-6	0.480±0.092 ^*^	0.321±0.056
6-11	1.070±0.132	0.811±0.082
11-16	1.771±0.162	1.582±0.159
16-21	1.768±0.208	2.255±0.439
21-26	1.792±0.418	1.554±0.440
26-31	0.990±0.165	1.345±0.345
31-36	0.247±0.504	0.015±0.128
36−41	−0.614±0.593	0.784±0.469
41−46	−0.348±0.311	−0.501±0.163

^*^ represents a significant difference (p<0.05) of corresponding traits between Ningchun 4 and ND646.

## Discussion

Transferring desirable genes from wheat wild relatives could be an efficient approach to broadening genetic diversity and boosting wheat genetic improvement (Qi et al., 2010). The creation of wheat-alien translocation lines is an efficient method to transfer desirable genes from wild relatives to common wheat compared with genetically unstable addition and substitution lines (Danilova et al., 2014). *H. californicum* has many potentially valuable traits, so it is vital to produce wheat-*H. californicum* translocation lines to transfer its useful genes to wheat for wheat improvement. A series of wheat-*H. californicum* addition lines was created. WJ28-1 is one of these lines and has been identified as DA6H^c^ by molecular markers (Fang et al., 2014). WJ28-1 showed several excellent traits, including disease resistance, more spikelets per spike, and more kernels per spike. Wheat translocation lines can be induced by spontaneous translocation, tissue culture, gametocidal chromosomes, *Ph*-systems, and ionization irradiation (Li et al., 2016). Therefore, WJ28-1 was irradiated by ^60^Co-γ to create translocation lines with small alien chromosome fragments. ^60^Co-γ irradiated WJ28-1 was backcrossed to Ningchun 4 for several generations to transfer the excellent alien genes from WJ28-1 into the Ningxia wheat background. A stable translocation line ND646 was developed during this process.

Determining chromosome constitutions is a crucial step in the introgression of elite genes into wheat. Sequential C-banding and GISH are extremely useful in identifying wheat-alien introgressions (Jiang and Gill, 1993). After the introduction of alien chromosomes into the wheat genome, sequential C-banding and GISH can be used to detect alien chromosomal fragments. However, repeated DNA probes are inefficient in identifying homoeologous chromosomes, as the abundance and distribution of repetitive elements vary among homoeologous chromosomes within species and chromosomes of closely related species (Badaeva et al., 1992; Friebe and Gill, 1993; Dedkova et al., 2007). PCR-based markers have been used extensively as effective tools for alien chromosome segment identification under wheat background (Wang et al., 2010; Zhuang et al., 2011; Zhao et al., 2014). In this study, ND646 was characterized by FISH and EST-PCR markers as a wheat-*H. californicum* 6H^c^S/6BL translocation line.

Wild relatives of wheat are valuable resources for expanding the gene pools of wheat breeding (Wu et al., 2006; Mullan et al., 2009). Although a large number of wheat-alien translocation lines carrying excellent alien genes have been produced, only a few have been successfully incorporated into wheat breeding programs. The development of translocation lines with lower linkage drag and regular meiotic behavior will increase the efficiency of using wild relatives in wheat breeding. In this study, ND646 carrying the short arm of *H. californicum* chromosome 6H^c^ exhibited many desirable traits, such as more kernels per spike, higher grain yields, higher sedimentation value, higher water absorption rate, higher hardness index, and resistance to powdery mildew, leaf rust, and 2,4-D. As a valuable germplasm for wheat breeding, ND646 will be continuously evaluated in regional trials of Ningxia and regional trials of the spring wheat region in northern China. Although the wheat 6BS chromosome was replaced by the 6H^c^S chromosome, it had a good compensatory effect, and ND646 showed excellent comprehensive agronomic traits. Due to whole 6H^c^ short arm translocation in ND646, the loss of alien chromosome fragments will inevitably occur in the process of inheritance, resulting in instability of the translocation line. To eliminate the genetic burden of large chromosome fragments, we will further improve ND646 using homoeologous recombination induced by the *Ph* mutant.
